# Physical activity and network attack tolerance preserve motor function in Parkinson’s disease: A pilot study

**DOI:** 10.1038/s41531-025-01033-9

**Published:** 2025-06-28

**Authors:** Adrian L. Asendorf, Elena Guerra, Verena Dzialas, Magdalena Banwinkler, Hendrik Theis, Niklas Hagemann, Kathrin Möllenhoff, Thilo van Eimeren, Merle C. Hoenig

**Affiliations:** 1 University of Cologne, Faculty of Medicine and University Hospital Cologne, Department of Nuclear Medicine, Cologne, Germany; 2 University of Cologne, Faculty of Mathematics and Natural Sciences, Cologne, Germany; 3 University of Cologne, Faculty of Medicine and University Hospital Cologne, Department of Neurology, Cologne, Germany; 4 University of Cologne, Institute of Medical Statistics and Computational Biology, Cologne, Germany; 5 Research Center Juelich, Institute for Neuroscience and Medicine II, Molecular Organization of the Brain, Juelich, Germany

**Keywords:** Parkinson's disease, Experimental models of disease, Functional magnetic resonance imaging, Magnetic resonance imaging, Positron-emission tomography, Network models

## Abstract

We tested whether network resilience, quantified by network attack tolerance (NAT), is associated with dopamine terminal (DaT) integrity, motor function and lifestyle factors in Parkinson’s disease (PD). Data from 22 individuals with PD and 39 healthy controls included information on lifetime physical activity (PA), cognitive/motor performance, putaminal DaT integrity, and resting-state fMRI. NAT was assessed at global and subnetwork level by calculating global efficiency upon iterative node removal. Linear-mixed-effects models were used to test the effects of PA, education, and dopamine integrity on NAT. Next, the moderating effect of lifestyle factors on the association between NAT and motor function were assessed, controlling for DaT integrity. Greater putaminal DaT integrity was linked to higher somatomotor NAT. Higher global and somatomotor NAT supported motor function, especially in patients with moderate lifetime PA. These preliminary results indicate that lifestyle factors serve network-specific attack tolerance, thereby aiding maintenance of motor function in early-stage PD.

## Introduction

PD is characterized by the progressive loss of striatal dopaminergic terminals (DaT), which is closely associated with the appearance of clinical features and can be quantified by single-photon emission computed tomography (SPECT). Importantly, the severity of cognitive and motor symptoms can vary significantly between individuals, despite similar levels of dopaminergic impairment. This points towards mechanisms that partially mitigate the effects of progressive loss of dopaminergic innervation and regional neurodegeneration on functional performance. These compensatory or coping mechanisms have been summarized under the umbrella term of *resilience* and can be facilitated by lifestyle factors such as lifetime physical activity (PA) or education. By definition, resilience can be divided into the concepts of motor reserve (MR) and cognitive reserve (CR). While MR is linked to the preservation of motor function, for instance, by the adaptation of motor-relevant networks, CR is associated with the relative maintenance of cognitive performance through the adaptation of cognitive-relevant networks^1,2^. To date, the neurobiological underpinnings of MR and CR in PD remain largely unknown.

Emerging evidence suggests improved efficiency, flexibility, and integration of functional networks to be associated with resilience in PD^3–6^. A metric that has recently been proposed to reflect CR in PD is network attack tolerance (NAT)^7^. NAT quantifies how well a network can maintain its structure and function when critical components of the network (i.e. nodes) are systematically removed^8^. Networks that continue to function well after these “attacks” are considered resilient. In the broader context of neurodegenerative diseases, NAT has more recently gained attention as a concept to characterize the brain’s actual compensatory capacity towards progressive neurodegeneration. In AD, NAT was shown to be altered along disease stages and cognitive performance levels, and has been proposed as a marker for CR, aiding the compensation for structural loss and the maintenance of cognitive performance^9,10^. In PD, recent findings suggest that higher NAT in the frontoparietal network is associated with the absence of cognitive decline in individuals with PD^7^, underscoring its relevance to CR in PD. However, it remains unknown whether NAT in PD is directly impacted by dopaminergic degeneration. Moreover, given that motor symptoms are among the most prominent clinical features of PD, it is yet to be determined whether greater NAT is also linked to the preservation of motor function (i.e., MR).

Notably, individual differences in resilience capacity, such as in MR or CR, have consistently been associated with certain lifestyle factors, such as education^11^ and PA^12^. In PD, education has been positively associated with CR^11,13,14^, while PA has traditionally been implicated as a proxy for MR. Both intense exercise interventions^15,16^ and higher lifetime/habitual PA^12,17^ have been demonstrated to attenuate motor decline and enhance dopamine availability^17,18^ (for a recent review see ref. ^19^). However, whether these lifestyle factors (i.e., reserve proxies) contribute to greater NAT remains unknown.

In this pilot study, we therefore aimed to address two objectives: (1) to examine the impact of putaminal DaT integrity and lifestyle factors (i.e., PA and education) on global and subnetwork NAT; and (2) to subsequently investigate the moderating effects of the lifestyle factors on the association between NAT and general motor and cognitive performance. These objectives were tested using cross-sectional data of individuals with early PD, who were part of the “Dopamine and Motor Control” (DoMoCo) study that encompasses an extensive array of lifestyle questionnaires, DaT SPECT, MRI imaging, and a detailed battery of cognitive and motor function tests. We hypothesized that preserved integrity of the dopaminergic system has a positive effect on NAT, particularly in the somatomotor network. We further hypothesized that, despite differences in dopaminergic impairment, lifetime PA (MR proxy) would be linked to greater NAT in the somatomotor network, whereas education (CR proxy) would be associated with greater NAT in cognition-relevant networks, such as the frontoparietal or attention network. Finally, we hypothesized that lifetime PA and education would moderate the association between NAT and general motor and cognitive performance, respectively.

## Results

### Effect of DaT integrity and reserve proxies on NAT

**Fig. 1:**
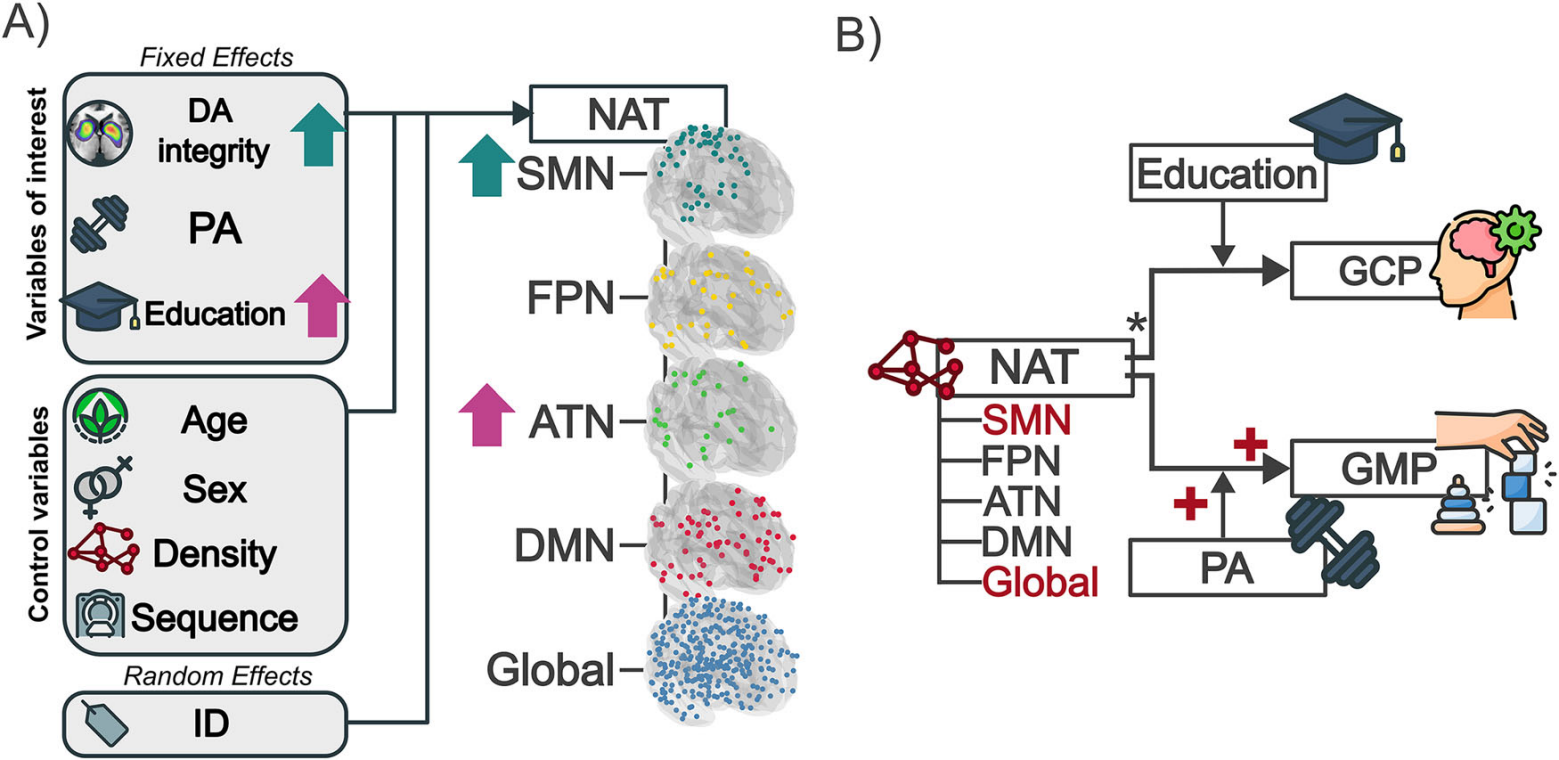
Model structure and key results. **A** Results of the generalized linear mixed models estimating the effect of biological (putamen dopamine terminal (DaT) integrity) and reserve factors (education and physical activity (PA)) on NAT. The arrows are color-coded and represent the direction of the association between NAT of the respective network and the covariate. The attention network (ATN) is shown in purple, and the somatomotor network (SMN) in turquoise. The surface projections depict the ROIs included for the somatomotor network (SMN, turquoise), the frontoparietal network (FPN, yellow), the attention network (ATN, green), the default mode network (DMN, red), and the global network (blue). **B** Results of the regression models. *Top:* Regression model predicting general cognitive performance (GCP) using education as a moderator. *Bottom:* Regression model predicting general motor performance (GMP) using PA as a moderator. The plus sign indicates a positive effect exerted by NAT (corresponding network also in red) and PA as a moderator on GMP. *Control variables for moderation analyses: age, sex, putaminal DaT, education or PA. For better visualization, effects of the control variables are not shown in both illustrations.

The generalized linear mixed-effects models (GLMMs) assessing the contributions of DaT integrity and reserve proxies on NAT, while controlling for demographic, network density and interscan variability, yielded a positive effect of putaminal DaT integrity (*β* = 0.079; *p* < 0.001) on somatomotor network (SMN) NAT (see Fig. 1). In addition, one of our reserve proxies, namely education, presented a positive effect (*β* = 0.013; *p* = 0.006) on NAT in the attention network (ATN) (i.e., higher education was associated with increased NAT in the ATN). Remaining models did not result in significant findings in terms of DaT integrity or reserve proxies.

Regarding the control variables, we found a positive effect of age (*β* = 0.001; *p* = 0.024), network density (*β* = 0.128; *p* < 0.001) and interscan variability (1 < 2: *β* = 0.035; *p* = 0.020, 1 < 3: *β* = 0.059; *p* = .026) on global NAT. For the SMN, there was a positive effect of density (*β* = 0.593; *p* < 0.001) and interscan variability (1 < 2: *β* = 0.109; *p* = 0.005) on NAT. In terms of default mode network (DMN) NAT, a positive effect of sex (*f* < *m*: *β* = 0.071; *p* = 0.043) and network density (β = 0.565; *p* < 0.001) was observed. Regarding ATN NAT, the model yielded a negative effect of sex (f < m. *β* = 0.071; *p* = 0.043) and a positive effect of network density. Finally, frontoparietal network (FPN) NAT was positively affected by network density (*β* = .671; *p* < .001) and interscan variability (1 < 2: *β* = 0.077; *p* = 0.014, 1 < 3: *β* = 0.198; *p* < 0.001). All results have been summarized in Table 1.

**Table 1:** Results table of GLMMs assessing the factors contributing to network attack tolerance (NAT) in five different networks

Fixed effects	Global				SMN				DMN				ATN				FPN			
	*ß*	*p*	CI		*β*	*p*	CI		*β*	*p*	CI		*β*	*p*	CI		β	*p*	CI	
Biological factor																				
Mean putamen	0.008	0.289	−0.007	.023	**0.079**	<0.001*	**0.040**	**0.118**	−0.030	0.144	−0.069	0.010	−0.010	0.531	−0.042	.022	0.014	0.395	−0.018	0.046
Reserve proxies																				
Physical activity	0.000	0.968	0.000	0.000	0.000	0.823	0.000	0.000	0.000	0.419	0.000	0.000	0.000	0.641	0.000	0.000	0.000	0.137	0.000	0.000
Education	−0.001	0.503	−0.006	0.003	−0.009	0.140	−0.020	0.003	0.004	0.444	−0.007	0.016	**0.013**	0.006*	**0.004**	**0.022**	0.001	0.815	−0.008	0.010
Control variables																				
Age	**0.001**	0.024*	**0.000**	**0.003**	−0.003	0.165	−0.007	0.001	−0.001	0.738	−0.005	0.003	0.001	0.686	−0.003	0.004	0.003	0.113	−0.001	0.006
Sex(f < m)	0.024	0.066	−0.002	0.051	−0.003	0.528	−0.090	0.046	**0.071**	0.043*	**−0.002**	**0.140**	**−0.069**	**0.015**	**−0.125**	**−0.014**	−0.025	0.373	−0.080	0.030
Network density	**0.128**	<0.001*	**0.119**	**0.138**	**0.594**	<0.001*	**0.570**	**0.618**	**0.565**	<0.001*	**0.544**	**0.586**	**0.754**	<0.001*	**0.722**	**0.786**	**0.671**	<0.001*	**0.645**	**0.697**
Sequence (1 < 2)	**0.035**	0.020*	**0.005**	**0.064**	**0.109**	0.005*	**0.033**	**0.185**	0.076	0.051	0.000	0.153	−0.008	0.795	−0.070	0.054	**0.077**	0.014*	**0.016**	**0.139**
Sequence (1 < 3)	**0.059**	0.026*	**0.007**	**0.110**	0.072	0.295	−0.063	0.207	0.087	0.209	−0.049	0.223	0.064	0.252	−0.046	0.174	**0.198**	<0.001*	**0.090**	**0.306**
Explained var.	R^2^ = 0.81 N = 198				R^2^ = 0.93 N = 198				R^2^ = 0.94 N = 198				R^2^ = 0.93 N = 198				R^2^ = 0.94 N = 198			

Significant findings are highlighted in bold.

*ß* unstandardized beta coefficients, *CI* represents the 95% confidence interval, *ATN* attention network, *DMN* default mode network, *FPN* frontoparietal network, *SMN* somatomotor network.

*p < 0.05.

No substantial differences in outcomes were observed when repeating the GLMMs in the smaller subgroup of patients only scanned with functional MRI (fMRI) sequence two. The results can be found in Supplementary Table 2. To further assess the potential influence of scanning sequence on our results, we calculated the temporal signal-to-noise ratio (tSNR) across all fMRI scans and sequences. The tSNRs were evenly distributed (See Supplementary Figure 2), indicating that our findings are unlikely confounded by systematic differences in signal quality across scanning sequences.

### Effect of NAT on motor function and cognitive function

Given that we observed an effect of DaT integrity and one reserve proxy, we next aimed to test whether global and subnetwork NAT has an impact on behavior, namely motor performance (GMP) and cognitive performance (GCP), while considering a moderating effect of our respective reserve proxy (PA and education). The results are described below.

In terms of GMP, the linear moderation models yielded GMP to be positively affected by higher global NAT (*β* = 279.127; *p* = .005) and SMN NAT (*β* = 117.415; *p* = .017), respectively. Remaining models did not yield a significant effect of subnetwork NAT on GMP. Moreover, we did not find a significant moderation effect of PA on this association, for SMN NAT (*β* = −1.418; *p* = .087) and global NAT (*β* = −2.536; *p* = .116). We further elucidated the moderation effects of PA on the NAT-GMP association using the Johnson and Neymann technique. The test showed a significant moderating effect of PA, which was only present at PA levels below a weekly energy expenditure score (WEE) of 46.66 for global NAT and 29.35 for SMN NAT (see Supplementary Figure 3). Hence, at similar NAT levels, participants with PD, who engaged in moderate PA demonstrated better GMP compared to those with a highly sedentary lifestyle, suggesting that a moderately active lifestyle, in contrast to a more sedentary one, may enhance the positive impact of NAT on motor performance (see Fig. 1B). In terms of our covariates, we observed a significant effect of education on GMP in the ATN model (*β* = −0.530; *p* = 0.033). Across the five tested moderation analyses, a negative effect of age on GMP was found (Global: *β* = −0.348; *p* < 0.001; SMN: *β* = −0.253; *p* = .002; DMN: *β* = −0.288; *p* = 0.002, ATN: *β* = −0.323; *p* < 0.001, FPN: *β* = −0.294; *p* = 0.003). The model outputs are summarized in Table 2.

**Table 2:** Results table of models assessing the interaction of network attack tolerance (NAT) and physical activity (PA) on motor performance (GMP) in five different networks

Effects on GMP	Global				SMN				DMN				ATN				FPN			
	*β*	*p*	CI		*β*	*p*	CI		*β*	*p*	CI		*β*	*p*	CI		*β*	*p*	CI	
Direct effects																				
NAT	**279.127**	0.005*	**102.564**	**455.69**	**117.415**	0.017*	**25.415**	**209.414**	43.665	0.417	−69.486	156.816	137.851	0.078	−18.204	293.907	68.816	.369	−91.791	229.423
PA	0.792	0.114	−0.219	1.802	0.345	0.083	−0.053	0.743	−0.017	0.901	−0.3	0.267	0.284	0.200	−0.1720	0.741	0.243	0.279	−0.224	0.709
Moderating effects																				
NAT × PA	−2.536	0.116	−5.793	0.721	−1.418	0.087	−3.075	0.238	0.105	0.864	−1.197	1.406	−1.335	0.208	−3.521	.851	−1.066	0.287	−3.153	1.02
Control variables																				
Education	−0.206	0.212	−0.545	0.134	−0.124	0.507	−0.517	0.27	−0.375	0.101	−0.836	0.086	**−0.53**	0.033*	**−1.007**	**−.052**	−0.265	0.230	−0.722	0.192
Mean putamen	0.115	0.851	−1.194	1.424	−0.402	0.629	−2.172	1.367	0.282	0.709	−1.326	1.891	0.513	0.484	−1.032	2.057	0.455	0.595	−1.359	2.27
** Age**	**−0.343**	<0.001*	**−0.481**	**−0.205**	**−0.253**	0.002*	**−0.39**	**−0.116**	**−0.288**	0.002*	**−0.449**	**−0.127**	**−0.323**	0.001*	**−0.477**	**−0.17**	**−0.294**	0.003*	**−0.463**	**−0.124**
Sex (*f* < *m*)	−0.700	0.483	−2.807	1.407	−0.335	0.773	−2.812	2.142	−0.239	0.86	−3.136	2.658	1.079	0.41	−1.675	3.834	0.315	0.803	−2.374	3.003
Sequence (1 < 2)	0.338	0.769	−2.115	2.791	0.748	0.577	−2.095	3.592	0.977	0.517	−2.211	4.166	1.751	0.184	−0.953	4.455	1.996	0.238	−1.502	5.493
Sequence (1 < 3)	−0.66	0.091	−8.003	0.683	−1.443	0.498	−5.945	3.059	−2.234	0.386	−7.643	3.175	−2.298	0.333	−7.257	2.662	−1.767	0.57	−8.357	4.823
Explained var.	*R*^2^ = 0.85 *N* = 22				*R*^2^ = 0.82 *N* = 22				*R*^2^ = 0.74 *N* = 22				*R*^2^ = 0.78 *N* = 22				*R*^2^ = 0.74 *N* = 22			

Significant findings are highlighted in bold.

*ß* unstandardized beta coefficients, *CI* represents the confidence interval, *ATN* attention network, *DMN* default mode network, *FPN* frontoparietal network, *SMN* somatomotor network.

*p < 0.05.

When repeating the linear regression models predicting GMP for individuals scanned with fMRI sequence two, the associations between global NAT (*β* = 272.154; *p* = 0.053) and SMN NAT (*β* = 101.601; *p* = 0.078) and GMP mentioned above did not reach significance. Interestingly, in the smaller group, ATN NAT had a significant, positive effect on GMP (*β* = 141.639; *p* = 0.048), which was moderated by lifetime physical activity (PA), particularly at lower PA levels (See Supplementary Table 3).

Concerning the association between GCP and NAT, none of the five linear moderation models demonstrated overall significance of the model test statistic, preventing interpretation of the individual effects of the moderation, and controlling variables.

Again, the same results were achieved when repeating the analysis in the smaller subgroup only including subjects scanned with fMRI sequence two.

## Discussion

In this study, we applied a multimodal neuroimaging approach (DaT SPECT, fMRI) combined with a detailed characterization of lifestyle, cognitive, and motor performance in a relative small group of 22 individuals with early PD. The overarching aim was to elucidate the role of a graph theoretical metric, namely NAT, as a potential motor and cognitive reserve measure that is linked to distinct lifestyle features. In this context, our key results were: (1) Lower putaminal DaT integrity was directly associated with decreased SMN NAT; (2) Higher SMN and global NAT were associated with better motor performance, regardless of the underlying dopaminergic impairment; (3) The positive effect of SMN NAT on motor performance was less pronounced in individuals with a relatively sedentary lifestyle (i.e., lower PA levels) compared to moderately active individuals. (4) Educational attainment asserted a direct effect, but no moderating effect, on NAT of a cognition-relevant network, namely the ATN. Taken together, the results suggest that NAT may represent a relevant resilience mechanism, which may compensate for the progressive loss of dopaminergic terminals in PD. Notably, as these findings may not readily extrapolate to moderate or advanced stages of the disease, all subsequent references to PD in the discussion pertain specifically to early-stage PD.

Importantly, and in line with our hypothesis, diminished DaT integrity was associated with lower SMN NAT. This observation was solely confined to the SMN, which contained several subcortical regions, including four regions of interest (ROIs) located within the basal ganglia. These results suggest that a diminished dopaminergic innervation of the basal ganglia may trigger a denervation within cortico-subcortical circuits of the SMN. This denervation or disconnection may result in decreased tolerance of the SMN towards consecutive perturbations. Decreased SMN NAT may in turn induce the clinical motor features of PD. Indeed, lower SMN and global NAT were associated with decreased motor performance independent of the level of DaT integrity. Overall, these findings expand on previous studies reporting dysfunctional connections to be primarily present between the dopamine-depleted putamen and the SMN^20,21^. Further, the results overall support the notion that the dopamine-dependent disruption of functional connectivity between the putamen and the SMN primarily fuels motor impairments in PD, as previously suggested^21^.

Notably, in the current study we focused on general motor performance rather than the severity of singular clinical features, such as tremor, rigidity, or akinesia. This is particular important concerning our investigations in terms of NAT being a potential MR mechanism. MR is defined as the relative preservation of motor function despite progressive neurodegeneration, through the functional adaptation of large-scale neural networks^2^. Here, we showed that independent of DaT loss, individuals with higher NAT in the global network and the SMN presented higher general motor performance. The current results expand on previous studies, which have mostly focused on regional structural^22^ or functional^4,5,23,24^ brain changes associated with MR. In contrast to these regional measures, NAT describes network properties that are more representative of the brain’s global ability to compensate against neurodegeneration. Our findings highlight that the ability to preserve an efficient information flow within global and motor processing areas (i.e., SMN) despite DaT loss leads to a relative preservation of general motor performance. NAT may thus form the basis for underlying MR mechanisms and represent a quantitative measure of MR capacity in PD.

Interestingly, in terms of cognitive function, we did not observe an effect of NAT in cognition-relevant networks on general cognitive performance, which contrasts findings from a recent study on the association of NAT and cognitive decline in PD^7^. Yet, the lack of findings in our cohort may be due to the absence of cognitive impairment in our studied cohort and thus, less variation in general cognitive performance. Moreover, it has consistently been shown that likelihood for cognitive decline increases in later stages of the disease^25^. Given that we focused on early PD, more refined assessments concerning NAT and cognition are necessary in cohorts with more advanced disease stages.

Importantly, despite the observed lack of association of cognitive function with NAT, we found that higher educational attainment, traditionally considered a cognitive reserve proxy, was positively associated with NAT of the ATN. This is in line with previous findings indicating that higher levels of education are associated with greater functional connectivity of the dorsal attention network^26^ and slower cognitive decline in healthy controls^27^. Interestingly, connectivity within the dorsal ATN appears to be crucial for cognitive performance as decreased connectivity in this network has been linked executive dysfunction in PD^28^. However, we neither found an association between ATN NAT and general cognitive performance nor a moderating effect of education in our PD cohort. Future assessments, in more advanced and clinically diverse PD stages may provide further insights into the role of educational attainment concerning a build-up of NAT and potential subsequent perseveration of cognitive performance. Moreover, education is a static, early lifetime factor, which may not entirely capture lifestyle-associated cognitive reserve mechanisms, as recently proposed^29^. More comprehensive measures, including composite lifestyle variables, may thus allow for more refined investigations of cognitive reserve aspects in PD.

While we observed a direct effect of education on NAT, we did not find any direct association of lifetime PA, our second lifestyle variable of interest with NAT. Importantly, lifetime PA and education were not correlated in our study cohort, suggesting an independent contribution of these factors to NAT and motor and cognitive reserve in PD. Indeed, we observed a positive moderating effect of PA on the association between 1) SMN NAT and general motor performance, and 2) global NAT and general motor performance. Specifically, at similar NAT levels, individuals with PD and highly sedentary lifestyles portrayed worse general motor performance compared to those with moderate PA levels. This suggests that a moderately active lifestyle, in contrast to a very sedentary one, may enhance the beneficial effects of NAT on motor performance. This is in line with other observations reporting a positive effect of premorbid PA on motor decline^12^, and an association between dopamine depletion and motor impairment^30^. One potential mechanism underlying this association could be the protective effect PA exerts on the basal ganglia’s structural integrity^31^, and the beneficial effect of PA on the basal ganglia’s dopaminergic innervation^17^. These effects may mitigate the severity of increasing striatocortical disconnection and thus aid the preservation of SMN NAT in the course of the disease^5,21^. Notably, in our study this protective effect was not observed for higher PA levels. This observation may be attributed to a recently reported U-shaped association of PA with motor performance in PD^30^, which needs to be validated in a cohort including more advanced disease stages. While several studies have already indicated that immediate, high-intensity exercise interventions positively impact PD symptomatology^15,16,18,19^, our results further demonstrate that also lifetime PA contributes to the build-up of MR-associated mechanisms, such as global and SMN NAT, that mitigate the effects of the disease later in life.

Several limitations need be considered when interpreting the current results. First, despite this well-characterized cohort, the sample was relatively small and replication in a larger sample is warranted. Nonetheless, the tested models explained a relatively high amount of variance. Moreover, given our repeated-measures design, 198 observations were included in the GLMMs that each estimated 8 coefficients, thereby increasing statistical power and reducing the risk of overfitting. Furthermore, the utilization of three different fMRI sequences may have potentially affected the results. To address this issue, we performed a sensitivity analysis in a subgroup of individuals scanned with the same sequence as well as a SNR assessment. Findings from these analyses suggested that scanner sequence had only a minimal influence on our findings. Aside from these statistical and technical drawbacks, we only considered physical activities between 20–49 years of age, thus potentially omitting contributions of early lifetime PA (12–19 years) to the build-up of reserve from that period. Finally, while our approach conceptualized education and lifetime PA as exclusive proxies for CR and MR, some studies show that these might be more inclusive. For example, PA has been shown to mediate the association between DaT integrity and cognition function^32^, while education levels have been shown to be linked with motor performance^33–35^. This asserts a potential value to assessing lifestyle factors in a more integrated way across cognitive and motor domains. Thus, advancing our understanding of how lifestyle features affect resilience mechanisms in PD may require a shift from investigating isolated lifestyle factors towards assessing lifestyle as a cumulative, integrated construct, encompassing multiple behavioral features.

Given the cross-sectional nature of this study and the focus on early disease stages, longitudinal study designs including more diverse patient populations at different stages of the disease (i.e. early to advanced), different PD subtypes (e.g., tremor-dominant vs. akinetic-rigid) and also a focus on non-motor, autonomic features of the disease will be pivotal to gain more insights into resilience mechanisms of the brain, such as SMN NAT. Identification of these factors and understanding how NAT might add to resilience mechanisms may overall provide novel insights regarding prevention and intervention strategies in PD.

Taken together, the results of this pilot study suggest that the brain’s compensatory capacity against neurodegenerative processes may be closely reflected by the degree of attack tolerance of distinct large-scale neural networks. Our findings underscore that lifelong behavioral factors, such as PA and education, affect the NAT of certain subnetworks, such as the somatomotor and attention network. Promoting these lifestyle factors may enhance the robustness of these networks, thereby contributing to the relative preservation of motor and cognitive performance despite the progressive loss of DaT in PD.

## Materials and methods

### Participants

This study comprised 22 individuals with early PD, who were recruited for the DoMoCo study during their clinical workup at the Department of Nuclear Medicine at the University Hospital Cologne, Germany. The DoMoCo study is conducted as part of the Collaborative Research Centre 1451 (CRC 1451) at the University Hospital Cologne. The inclusion criteria for this study were: 1) age between 49 and 80 years; 2) early-stage PD (clinical symptoms < 3 years); 3) available in-house DaT SPECT imaging; 4) right-handedness; 5) absence of severe cognitive deficits (Montreal Cognitive Assessment (MoCA) score ≥ 24); 6) absence of depressive symptoms (Geriatric Depression Scale score *≤* 5); 7) no current administration of selective serotonin reuptake inhibitors; 8) absence of significant other neurological disorders; 9) MRI safe (e.g. no pacemaker). Notably, all tests and imaging procedures in the DoMoCo study PD were performed in participants with PD, while they were off medication. As 13 of the 22 patients had already received dopaminergic medication, they were asked to discontinue their medication prior to study participation. This represented at least 12 h of withdrawal of dopamine replacement therapy or 72 h for extended release of dopamine agonists.

A group of 39 healthy controls, who underwent the study protocol of the DoMoCo study, except the DaT SPECT imaging, was used as reference for the cognitive and motor tests.

The study was approved by the ethics committee of the University Clinic of Cologne (reference: 20–1422) and was conducted according to the standards of the Declaration of Helsinki. All participants gave informed consent prior to enrollment and received monetary compensation for their participation. Demographic characteristics are summarized in Table 3.

**Table 3:** Demographics of the study cohort

	Mean (SD) /Median (IQR)	
	PD (*n* = 22)	HC (*n* = 39)
Age	62.1 (8.0)	63.5 (6.5)
Sex (*m*/*f*)	15/7 *m* = 68%	26/13 *m* = 50%
Putaminal DaT integrity (z-scores)	−3.6 (0.7)	NA
Education (years)	15.8 (2.7)	16.7 (2.8)
PA	58.3 (82.8)	48.1 (34.0)
MoCA	27 (2)	28 (2)
GCP (z-scores)	−0.4 (2.4)	NA
GMP (z-scores)	−3.2 (3.1)	NA
MDS UPDRS III	14 (6)	0 (1)
LEDD	206.4 (113.2)	NA

Standard deviation (SD) is provided for continuous variables and interquartile range (IQR) for ordinal values (MDS UPDRS-III and MoCA scores). While the z-scores for DaT integrity were derived from an internal software cohort, the z-scores for GCP and GMP were based on the 39 HCs described in this table.

*PD* Parkinson’s disease, *HC* healthy control, *DaT* dopamine transporter, *PA* lifetime physical activity, *MoCA* Montreal Cognitive Assessment, *GCP* general cognitive performance, *GMP* general motor performance, *MDS UPDRS-III* Movement Disorder Society Unified Parkinson’s Disease Rating Scale-Part III, *LEDD* Levodopa equivalent daily dose.

### Reserve proxies—lifetime physical activity and education

Lifetime PA was used as a proxy for MR and assessed using the Historical Leisure Activity Questionnaire (HLAQ)^36^ and metabolic equivalents (METs)^37^, which were assigned to account for the intensity of the respective activity. The HLAQ consists of four periods (12–19 years, 19–35 years, 35–50 years, above 50 years). For each period, the participant had to indicate which activity he or she performed more than 10 times and specify the number of hours per week, months per year, and years per period. Using the formula below (see Formula 1), we first calculated the average weekly number of hours each activity was carried out per period. Next, we obtained the weekly energy expenditure for each activity by multiplying the average weekly hours per activity by the corresponding MET score. Finally, a total weekly energy expenditure score (WEE_period_) was calculated over all activities reported in that period. In this study, lifetime PA was defined as the mean of the WEE_period_ calculated for the periods 19–35 years and 35–50 years. We excluded the earliest period of the questionnaire (i.e., 12–19 years), given that a recall bias was noticed across participants. We also excluded the latest period (i.e., above 50 years) because all patients were only recently diagnosed with PD at around 50 years of age, which may have influenced their physical activity performance.

1 $${{WEE}}_{{period}}=\mathop{\sum }\limits_{i=1}^{{n}_{{period}}}\frac{{{years}}_{i}* {{month}}_{i}* {{weekly\; hours}}_{i}}{12* {{years}}_{{period}}}* {{MET\; score}}_{i}$$

Aside from the lifetime PA variable, we further used educational attainment as proxy for CR. Educational attainment was defined as the total number of years spent in formal schooling, vocational training and/or university education.

### Behavioral assessment—cognitive and motor function

All participants in the DoMoCo study underwent a detailed clinical, motor, and cognitive assessment. Clinically, disease severity was evaluated using the Movement Disorder Society Unified Parkinson’s Disease Rating Scale (MDS-UPDRS) Part III^38^. To obtain more quantitative measures for cognitive and motor performance that go beyond clinical severity ratings, a battery of motor and cognitive tests was included in the current analysis. The test battery was conducted in the context of the Human Motor Assessment Centre (CRC 1451) and consisted of the following tests: Motor function was evaluated by measuring maximum grip strength and through the Finger Tapping Test, Purdue Pegboard Test, Jebsen-Taylor Hand Function Test, and Timed Up and Go Test. With exception of the Timed up and Go Test^39^, a detailed description of the motor battery can be found elsewhere^40^. The cognitive battery consisted of the Cologne Neuropsychological Screening Tool for Stroke^41^, DemTect Test^42^ and Trail Making Test^43^. To obtain a measure for general motor performance (GMP) and general cognitive performance (GCP), the performance scores of the motor and the cognitive test battery were z-standardized using the sample of 39 age- and sex-matched healthy controls as reference. Subsequently, these z-scores were combined into two single composite scores, GMP and GCP, respectively. As longer durations in the Jebsen-Taylor, Timed Up and Go, and Trail Making Test represented poorer performance, these test scores were inverted prior to the composite score calculation.

### Dopamine transporter SPECT acquisition and preprocessing

DaT SPECT images were acquired on a PRISM-3000 three-head SPECT system (Philips/Picker) located at the University Hospital of Cologne, following a standardized clinical procedure. The EARL-BRASS software (Hermes, Sweden^44^), was used for reconstruction and quantification of the DaT SPECTS, including attenuation correction^45^ and spatial normalization to a reference sample of age-matched healthy controls, resulting in z-transformed deviation maps. Finally, for the current analysis, mean z-values of the bilateral putamen were extracted from these maps using EARL-BRASS. As the SPECT system used did not include additional CT or MRI imaging, correction for partial volume effects was not feasible. Nonetheless, this method has consistently been shown to capture dopaminergic integrity necessary for a reliable diagnosis of Parkinson’s disease^46–48^.

### fMRI acquisition

All participants underwent a MRI session at the Research Centre Juelich. Anatomical T1-weighted MRI and resting state (rs) fMRI series were acquired on a 3 T Siemens Prisma scanner. The anatomical sequence followed a specific protocol: repetition time (TR) = 2300 ms, echo time (TE) = 2.32 ms, flip angle = 8°, slice thickness = 0.90 mm, voxel size = 0.9 × 0.9 × 0.9 mm, number of slices = 192. For each individual, one of three different fMRI sequences was available. Four individuals were scanned with sequence one: TR = 800 ms, TE = 37 ms, multiband SMS factor = 8, flip angle = 52°, slice thickness = 2 mm, voxel size = 2.0 × 2.0 × 2.0 mm, number of volumes 740, acquisition time = approx. 10 min. While 17 individuals were scanned with sequence two: TR = 980 ms, TE = 30 ms, multiband SMS factor = 4, flip angle = 70°, slice thickness = 2.2 mm, voxel size = 2.2 × 2.2 × 2.2 mm, number of volumes 500, acquisition time = ~8 min. Finally, one individual was scanned with sequence three: TR = 1120 ms, TE = 57 ms, multiband SMS factor = 8, flip angle = 52°, slice thickness = 2 mm, voxel size = 2.0 × 2.0 × 2.0 mm, number of volumes 740, acquisition time = ~13 min. The sequence changes were unintended, but necessary due to an unforeseen, minor update of the scanner’s software during the study period.

### fMRI preprocessing

Preprocessing of the fMRI data was carried out using the CONN toolbox v20.b (Whitfield-Gabrieli & Nieto-Castanon, 2012) implemented in Matlab (Matlab R2020b Update 4, MathWorks, Inc., Natick, Massachusetts, United States). CONN’s default preprocessing pipeline was used to normalize the images to MNI (Montreal Neurological Institute) space (web.conn-toolbox.org/fmri-methods/preprocessing-pipeline). The pipeline included spatial realignment, slice-timing correction, outlier identification, segmentation, normalization, and smoothing (Gaussian kernel 8 mm full-width half maximum). The preprocessing was performed separately for each of the three sequences.

### Graph theoretical network construction and network attack

In graph theory, the brain is conceptualized as a network of nodes (regions) connected by edges (functional connections). NAT is assessed by systematically deleting nodes (i.e., attacks) from this network and evaluating whether the network sustains efficient communication. Concomitantly, networks that maintain their efficiency despite successive attacks represent high NAT. How efficiently information is exchanged in a network can be estimated by global efficiency (GE), which assesses the shortest path length between all pairs of nodes in a network^49,50^. Importantly, unlike other graph theoretical metrics, GE can account for paths of disconnected nodes, rendering it especially suitable for disconnected networks^8,51^ and hence, a robust measure of efficiency for NAT.

In a first step, we used a set of 300 predefined, spherical, non-overlapping ROIs^52^ to compute individual ROI-to-ROI connectivity matrices in CONN. These 300 × 300 covariance matrices represent the functional connectivity between each ROI pair. Each element or tile of these covariance matrices contained the respective Fisher-transformed bivariate correlation coefficient between the Blood Oxygen Level-Dependent time series of two ROIs.

**Fig. 2:**
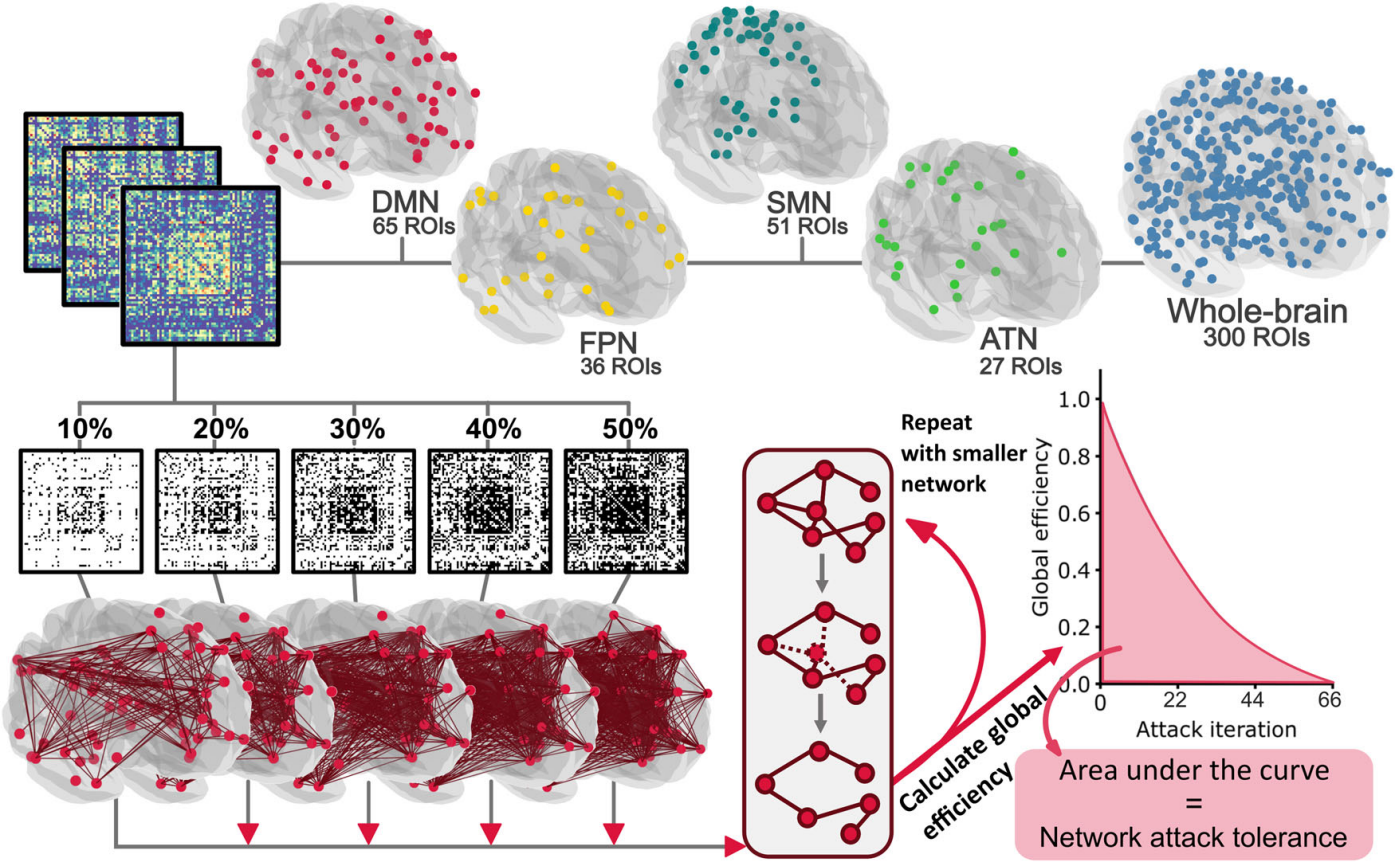
Illustration of the analysis pipeline. Top**:** Individual resting-state connectivity matrices were computed using a Region of interest (ROI) based functional atlas. ROIs of the attention network (ATN, green), the default mode network (DMN, red), the frontoparietal network (FPN, yellow), the somatomotor network (SMN, turquoise) and the whole-brain network (blue) were analyzed separately. The analysis steps are illustrated using the DMN as an example. Bottom left: Connectivity matrices were thresholded at nine different densities from 10% to 50% (for clarity only five out of nine densities are depicted above), then binarized, and finally used for network construction. Bottom right**:** During the attack analysis, the network’s nodes were iteratively removed in descending order of their connectedness. For each removed node, the network’s global efficiency was computed. The ability to sustain its global efficiency to successive attacks (area under the curve) defined the attack tolerance of the respective network. All renderings and covariance matrices were plotted using nilearn^64^.

In a second step, we computed weighted, undirected, and unthresholded graphs based on the covariance matrices utilizing the Brain Connectivity Toolbox (brain-connectivity-toolbox.net)^8^ and the Graph Analysis Toolbox (cbrain.stanford.edu/Tools.html)^53^. These graphs were computed on the whole-brain (global) level as well as the single subnetwork level. The single subnetworks of interest were the DMN (65 ROIs), the FPN (36 ROIs), the SMN (51 ROIs), and the ATN (27 ROIs), which are the major large-scale cognitive and motor networks defined by various resting state studies^54–56^. Importantly, none of these subnetworks included overlapping ROIs. The key regions for each network are visualized in Fig. 2 and listed in Supplementary Table 1. Subsequently, each of these graphs was proportionally thresholded, preserving only the strongest ten to fifty percent of the weighted edges in the graph, respectively. We chose this broad range as there is no consensus on appropriate thresholds for NAT analyses. Consequently, by using five percent increments, nine different network densities per network were examined. Each of these graphs was binarized, leaving nine differently thresholded, unweighted, undirected graphs per network and individual. Fig. 2 provides a graphic outline of the analysis and Supplementary Figure 1 portrays the approach as a detailed procedural map. In a final step, the constructed graphs were attacked using the same set of toolboxes. During the attack analysis, nodes were iteratively removed from the network in descending order of their degree (i.e., the number of edges connected to that node). Thus, nodes with the most connections were removed first. After each node removal, the network’s GE was calculated (see Formula 2). GE was defined by the average inverse shortest path length between all pairs of nodes in the network, where N represented the number of nodes in the network and L_ij_ was the shortest path between nodes i and j^49^:

2 $${GE}=\frac{1}{N\left(N-1\right)}\sum _{i,j,j\ne 1}\frac{1}{{L}_{{ij}}}$$

GE was then plotted on a graph for each attack iteration (see Fig. 2). The area under the curve (AUC) of each graph was used to obtain one single measure of individual NAT. Next, the AUC was normalized according to the number of attacks performed (i.e., the number of nodes in the respective network). After normalization, NAT described how many percent of the maximum possible GE was retained during attacks.

### Statistical analysis: generalized linear mixed-effects models and moderation analysis

In a first step, we wanted to examine the direct effects of two reserve proxies, specifically lifetime PA and education, as well as the effects of dopaminergic impairment, namely putaminal DaT integrity, on NAT, while accounting for individual variances in demographics. To account for the repeated measure design due to the different network densities, we performed GLMMs^57^, allowing individual intercepts for each participant. Fixed effects included lifetime PA, education, and putaminal DaT-integrity, while controlling for network density, fMRI sequence (interscan variability), age, and sex. Since NAT is a fraction and by design never one or zero, a beta regression model was used^58^, i.e., a GLMM with a beta response distribution utilizing a logit link function. R-squared estimates of the GLMMs were derived from the ‘mgcv’ R package^59^. The significance level was set to *p* = 0.05.

Next, we assessed the potential moderating effects of lifetime PA and education on the association between NAT and motor/cognitive performance. To do this, we ran two linear moderation models estimating either general motor or cognitive performance based on the respective network’s NAT, the reserve proxies (education and PA) as well as their interaction. Since NAT was originally a repeated measurement (due to the nine different network densities), we used the mean NAT value across all densities for each network as input variable. More specifically, in the first model we estimated motor performance (GMP) using putaminal DaT integrity, age, sex, fMRI sequence, education, and NAT, introducing PA as moderating variable. The second model estimated cognitive function (GCP) using putamen DaT integrity, age, sex, fMRI sequence, PA, and NAT as covariates and education as a moderating variable. Since GCP was missing for one individual this model was run on data of 21 individuals. Finally, the Johnson-Neyman technique^60^ was used to identify ranges of significance and visualize interaction effects in the data.

All statistical models listed above were run separately for global and subnetwork NAT (5 runs) using R (Rstudio ver. 2022.12.0) and the following packages: ‘mgcv’^59^, ‘ggplot2’^61^, ‘interactions’^62^, ‘broom’^63^. To evaluate the impact of fMRI sequence variability on our results, we re-ran all statistical analyses in a subgroup of 17 individuals with PD, who were exclusively scanned with fMRI sequence two.

## Supplementary information

Supplementary Information

## Data Availability

The data supporting this study’s findings are findable in the CRC1451 data registry (https://www.crc1451.uni-koeln.de/), and reasonable requests can be addressed to the corresponding author.

## Code availability

The underlying inhouse code for this analysis is available on GitHub and can be accessed via this link https://github.com/Asendorfa/NAT. We provide a procedural map in the supplements that depicts which script was used for which analysis step (See Supplementary Figure 1).
