# Supplementary material for: Physical activity and network attack tolerance preserve motor function in Parkinson’s disease: A pilot study
Source: NPJ Parkinsons Dis. 2025 Jun 28;11:183. doi: 10.1038/s41531-025-01033-9 (PMC12206233; doi:10.1038/s41531-025-01033-9)
Supplement: Supplementary file 1 — Supplementary Information [file 41531_2025_1033_MOESM1_ESM.pdf]

Supplementary Material:

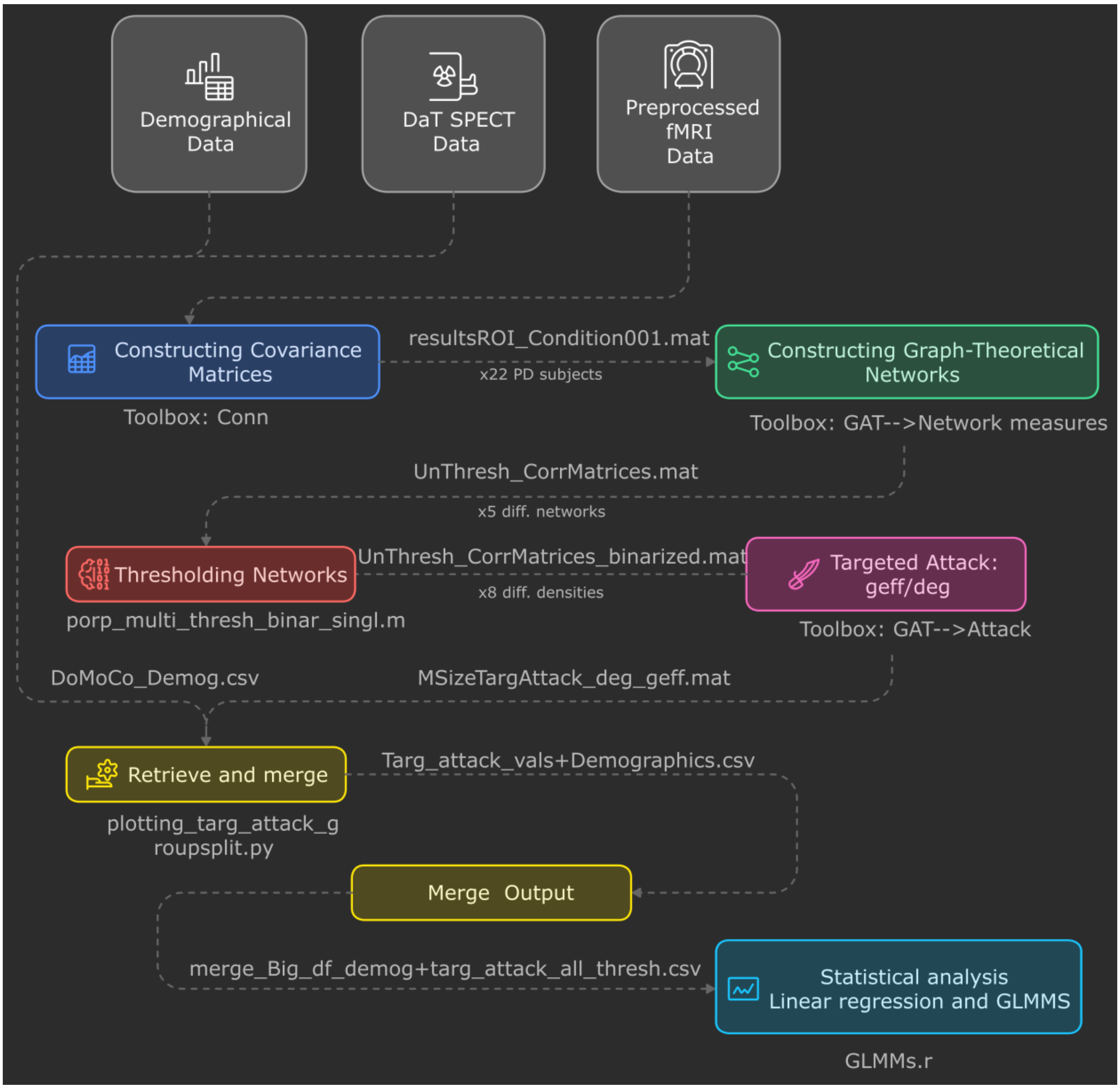

Supplementary Figure 1: Procedural map of the analysis pipeline implemented. Grey boxes represent the data that served as an input to the pipeline. The colored boxes represent each step undertaken throughout the analysis. Underneath each box, the toolbox or script used for this step is depicted. The text above the arrows indicates the data format generated as an output and subsequently served as an input in the next step. If this step was carried out multiple times, it is mentioned below the arrow.

Functional Network	AAL_Labels
SMN<br>(51 ROIs)	Precuneus_L, Cingulum_Mid_L, Parietal_Inf_L, Postcentral_L, Precentral_L, Postcentral_L, Precentral_L, Postcentral_L, Postcentral_L, Postcentral_L, Parietal_Sup_L, White Matter ROI, Paracentral_Lobule_L, Paracentral_Lobule_L, Paracentral_Lobule_L, Paracentral_Lobule_R, Supp_Motor_Area_R, Cingulum_Mid_R, Precuneus_R, Supp_Motor_Area_R ,Postcentral_R, White Matter ROI , Postcentral_R, Precentral_R, Postcentral_R ,Precentral_R ,Precentral_R , Precentral_R, Postcentral_R, Postcentral_R, Frontal_Sup_R, SupraMarginal_R, Putamen_R, Putamen_L, Thalamus_R, Thalamus_L, Cerebelum_7b_L, Cerebelum_8_R, Cerebelum_4_5_L, Cerebelum_4_5_R, Postcentral_L, Postcentral_L, Insula_R, Postcentral_R, Postcentral_R, Pallidum_R, Putamen_L, Thalamus_R, Thalamus_L, Cerebelum_6_L, Cerebelum_6_R
DMN<br>(65 ROIs)	Temporal_Mid_L, Temporal_Mid_L, Temporal_Mid_L, Temporal_Mid_L, Temporal_Mid_L, Temporal_Mid_L<br>Temporal_Mid_L, Frontal_Inf_Orb_L, Angular_L, Temporal_Pole_Mid_L, Occipital_Mid_L, Angular_L, Frontal_Mid_L, Frontal_Sup_L, Frontal_Sup_L, Frontal_Sup_Orb_L, Frontal_Sup_L, Lingual_L, Precuneus_L, Frontal_Sup_Medial_L, Frontal_Sup_L, Cingulum_Ant_L, Frontal_Sup_Medial_L, Precuneus_L, Cingulum_Ant_L, White Matter ROI , Cingulum_Mid_L, Frontal_Med_Orb_L, Frontal_Sup_Medial_L, Cingulum_Ant_L, Frontal_Med_Orb_R, Precuneus_R, Frontal_Sup_Medial_R, Frontal_Sup_Medial_R, Cingulum_Post_R, Frontal_Med_Orb_R, Rectus_R, Frontal_Sup_Medial_R, Precuneus_R, Frontal_Sup_Medial_R, Frontal_Sup_Medial_R, Cingulum_Ant_R, Precuneus_R, Frontal_Sup_R, Frontal_Sup_R, Occipital_Mid_R, Temporal_Pole_Mid_R, Angular_R, Frontal_Inf_Orb_R, Angular_R, Temporal_Mid_R, Temporal_Mid_R, Temporal_Mid_R, Temporal_Mid_R, Fusiform_L ,Hippocampus_L, Hippocampus_R, Thalamus_R,m Thalamus_L, Cerebelum_Crus2_L, Cerebelum_Crus2_R, Cerebelum_Crus1_L, Cerebelum_Crus1_R, Cerebelum_9_L, Cerebelum_9_R,
ATN<br>(27 ROIs)	Occipital_Sup_L, Fusiform_L, Temporal_Inf_R, Cingulum_Mid_R, Temporal_Mid_L, Temporal_Inf_L, Parietal_Inf_L, Precentral_L, Precuneus_L, Precuneus_R, Occipital_Sup_R, Parietal_Sup_R, Frontal_Mid_R, Temporal_Mid_R, Cerebelum_9_L, Cerebelum_9_RTemporal_Mid_R, Temporal_Mid_L, Temporal_Sup_L, Temporal_Sup_R, Temporal_Sup_R, Frontal_Inf_Tri_L, Supp_Motor_Area_L, Frontal_Inf_Tri_R, Temporal_Sup_R, Putamen_L, Putamen_R
FPN<br>(36 ROIs)	Parietal_Inf_L, Frontal_Inf_Oper_L, Precentral_L, Frontal_Mid_L, Angular_L, Frontal_Inf_Tri_L, Precentral_L, Frontal_Mid_Orb_L, Frontal_Mid_L, Parietal_Inf_L, Frontal_Sup_L, Frontal_Sup_Medial_L, Frontal_Sup_Orb_R, Frontal_Mid_R, Angular_R, Frontal_Mid_Orb_R, Angular_R, Frontal_Mid_R, Frontal_Mid_R, Parietal_Inf_R, Frontal_Mid_Orb_R, Precentral_R, Frontal_Inf_Tri_R, Parietal_Inf_R, Temporal_Inf_R, Frontal_Inf_Tri_R, SupraMarginal_R, Caudate_R, Caudate_L, Cerebelum_Crus1_L, Cerebelum_Crus1_R, Cerebelum_Crus2_L, Cerebelum_7b_R, Cerebelum_Crus1_L, Cerebelum_6_R, Cerebelum_Crus1_R

Supplementary Table 1: Overview of AAL regions assigned to each ROI of each functional subnetwork. The table lists all included AAL labels, grouped by functional network and summarized by broader anatomical-functional categories. White Matter ROI =relates to ROIs located outside the of AAL atlas. AAL = Automated Anatomical Labeling; L = left; R = right; Sup = superior; Mid = middle; Inf = inferior; ROI = region of interest; SMN = somatomotor network; DMN = default mode network; FPN = frontoparietal network; ATN = attention network.

Fixed effects	Global				SMN				DMN				ATN				FPN			
	ß	*p*	CI		β	*p*	CI		β	*p*	CI		β	*p*	CI		β	*p*	CI	
Biological factor																				
Mean putamen	.010	.194	*-.005*	*.024*	**.086**	**<.001***	***.042***	***.129***	-.028	.238	*-.075*	*.019*	-.017	.389	*-.055*	*.022*	.019	.328	*-.019*	*.056*
Reserve proxies																				
Physical activity	.000	.525	*.000*	*.000*	.000	.760	*.000*	*.000*	.000	.312	*-.001*	*.000*	.000	.710	*.000*	*.000*	.000	.155	*.000*	*.001*
Education	.000	.503	*-.004*	*.004*	-.005	.464	*-.017*	*.008*	.006	.393	*-.008*	*.019*	**.013**	**.028***	***.001***	***.023***	.000	.947	*-.010*	*.011*
Control variables																				
Age	**.001**	**.073***	***.000***	***.003***	-.004	.128	*-.008*	*.001*	-.001	.682	*-.006*	*.004*	.000	.958	*-.004*	*.004*	.003	.068	*.000*	*.007*
Sex(f<m)	.0064	.067	*-.020*	*.031*	-.006	.144	*-.133*	*.019*	**.049**	**.236***	***-.032***	***.132***	**-.079**	**.015**	***-.147***	***-.012***	-.005	.881	*-.070*	*.060*
Network density	**.115**	**<.001***	***.105***	***.124***	**.581**	**<.001***	***.552***	***.610***	**.572**	**<.001***	***.548***	***.597***	**.778**	**<.001***	***.745***	***.812***	**.685**	**<.001***	***.655***	***.715***
Explained var.	R^2=.83 N=153				R^2=.93 N=153				R^2=.94 N=153				R^2=.95 N=153				R^2=.94 N=1538			

Supplementary Table 2: Results table of GLMMs assessing the factors contributing to network attack tolerance (NAT) in five different networks only in patients assessed with sequence 2. Significant findings are highlighted in bold. ß = unstandardized beta coefficients, *: p<.05, CI represents the 95% confidence interval, ATN = attention network, DMN = default mode network, FPN = frontoparietal network, SMN =somatomotor network

Effects on GMP	Global				SMN				DMN				ATN				FPN			
	β	p	CI		β	p	CI		β	p	CI		β	p	CI		β	p	CI	
Direct effects																				
NAT	272.154	.053	-4.605	548.913	101.601	.078	-13.922	217.124	6.737	.903	-114.812	128.286	**141.639**	**.048***	**1.585**	**281.692**	77.301	.309	-85.009	239.611
PA	.839	.192	-.508	2.187	.348	.137	-.135	.831	-.058	.665	-.349	.234	.395	0.056	-0.013	0.803	.105	.638	-.384	.594
Moderating effects																				
NAT x PA	-2.688	.194	-7.025	1.649	-1.431	.141	-3.435	.572	.280	.646	-1.053	1.613	-1.879	.058	-3.837	.080	-.466	.640	-2.647	1.715
Control variables																				
Education	-.259	.172	-.655	.136	-.155	.433	-.583	.273	-.321	.176	-.815	.173	-.418	.056	-.847	.012	-.255	.257	-.730	.221
Mean putamen	-.067	.921	-1.569	1.434	-.413	.651	-2.41	1.583	.175	.827	-1.584	1.935	.764	.279	-.736	2.264	.037	.966	-1.865	1.938
**Age**	**-.348**	**0.001***	**-.505**	**-.191**	-.291	.002*	-.449	-.133	-.31	.003*	-.486	-.134	**-.346**	**>0.001***	**-.490**	**-.202**	**-.34**	**.002***	**-.523**	**.157**
Sex (f<m)	-.738	.51	-3.173	1.697	-1.04	.447	-4.000	1.921	-.89	.537	-4.027	2.247	-.346	.780	-3.066	2.374	-.553	.670	-3.39	2.284
Explained var.	R^2=.80 N=17				R^2=.79 N=17				R^2=.71 N=17				R^2=.78 N=17				R^2=.73 N=17			

Supplementary Table 3: Results table of models assessing the interaction of network attack tolerance (NAT) and physical activity (PA) on motor performance (GMP) in five different networks only in patients assessed with sequence 1. Significant findings are highlighted in bold. ß = unstandardized beta coefficients, *: p<.05, CI represents the confidence interval, ATN = attention network, DMN = default mode network, FPN = frontoparietal network, SMN =somatomotor network. DMN (grey) regression model itself did not reach level of significance, but is reported for transperency

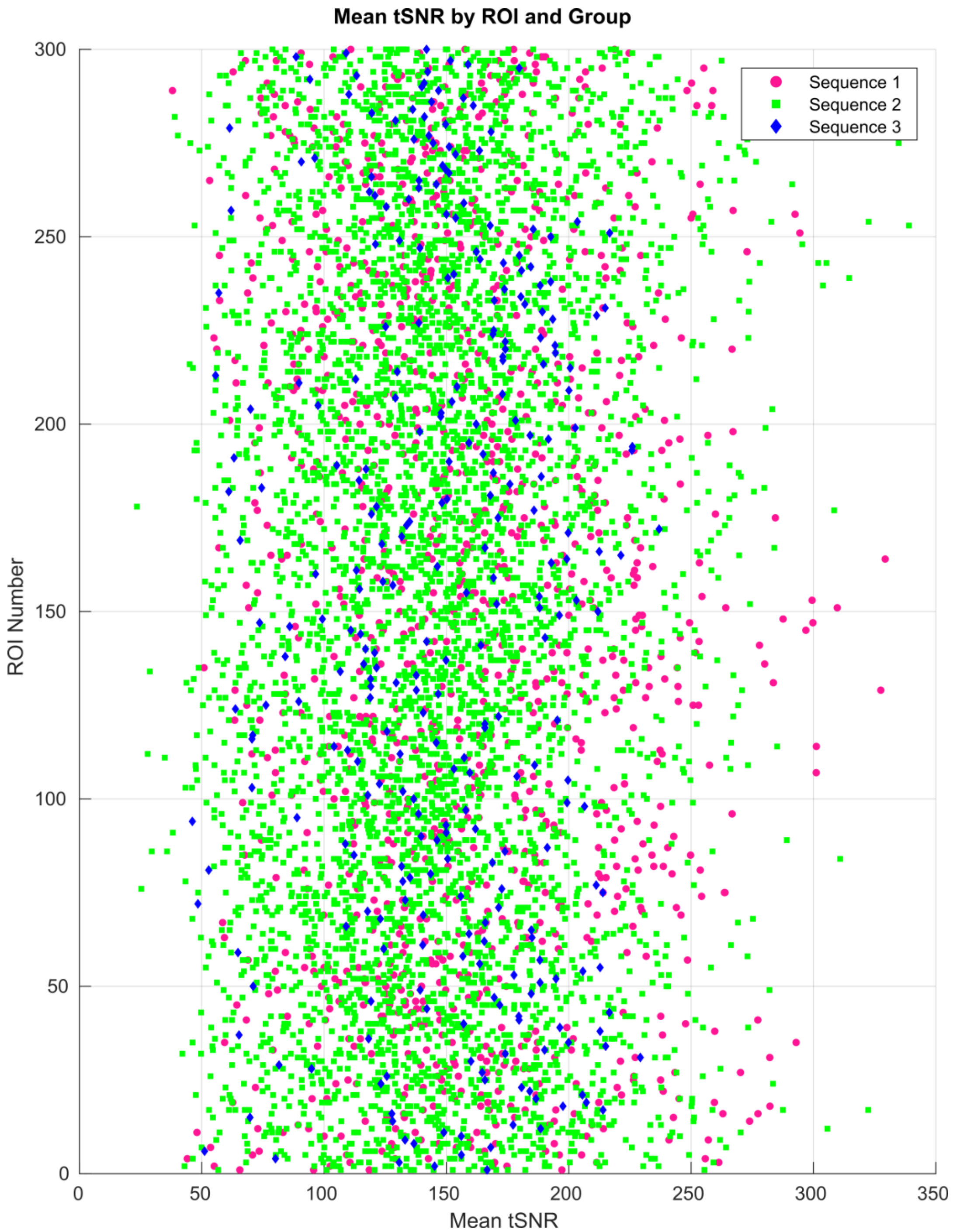

Supplementary Figure 2: Temporal signal-to-noise ratio of each ROI across patients grouped by scanning sequence. Each dot represents the average temporal signal-to-noise ratio tSNR of an individual region of interest (ROI) of one patient, color-coded by sequence: pink for sequence 1, green for sequence 2, and blue for sequence 3. The tSNR maps were computed for each patient as the mean signal divided by the standard deviation across time for each voxel. These tSNR maps were then used to calculate the mean tSNR for each individual ROI included in the 300 ROI Seitzman atlas.

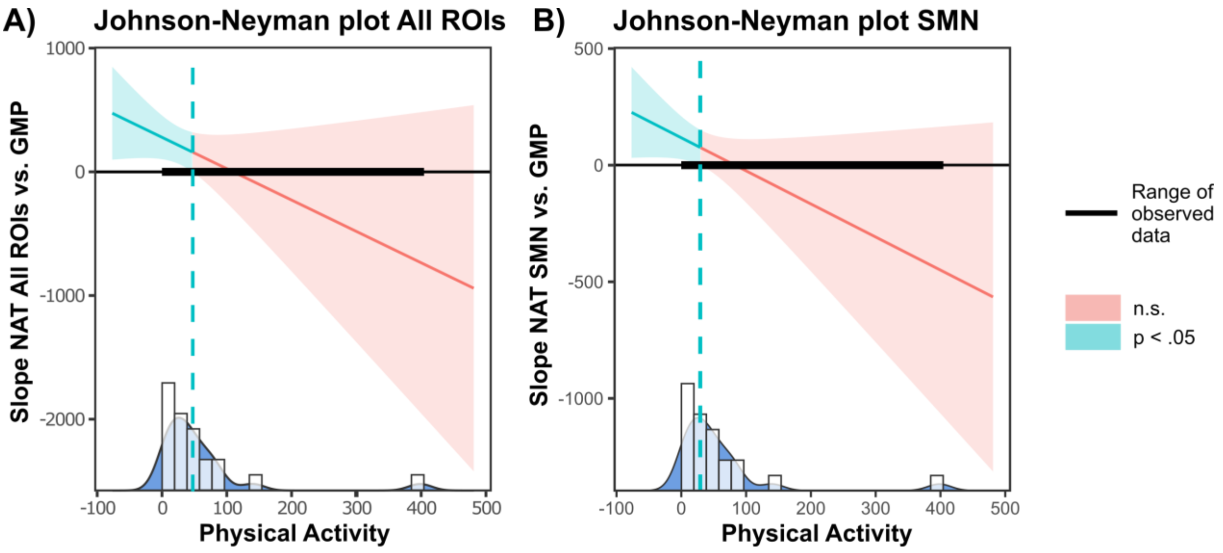

Supplementary Figure 3: Johnson-Neyman plots. Johnson and Neyman plots assessing the moderating effect of PA on the association between general motor performance (GMP) and global network attack tolerance (NAT) (A) and the association between GMP and somatomotor network (SMN) NAT (B). Ranges where PA significantly moderated the effect on the association between NAT and GMP are shown in light blue. Ranges of non-significance are depicted in red. The bold black bar indicates the range of observed data. On the x axis a 'densigram' (density in dark blue and histogram in white) shows the distribution of observed PA levels, providing context for the applicability of the identified ranges of significance. Significance threshold: $p < .05$.

Link to Pictograms used:

Cognitive performance: https://www.freepik.com/icon/cognitive-function_11604264

Motor function: https://iconscout.com/icon/fine-motor-skills-5149837

STROBE statement: Reporting guidelines checklist for cohort, case-control and cross-sectional studies

SECTION	ITEM NUMBER	CHECKLIST ITEM	REPORTED ON PAGE NUMBER:
**TITLE AND ABSTRACT**			
	1a	Indicate the study's design with a commonly used term in the title or the abstract	0-1
	1b	Provide in the abstract an informative and balanced summary of what was done and what was found	1
**INTRODUCTION**			
Background and objectives	2	Explain the scientific background and rationale for the investigation being reported	1-2
	3	State specific objectives, including any pre-specified hypotheses	3-4
**RESULTS**			
Participants	13a	Report numbers of individuals at each stage of study—eg numbers potentially eligible, examined for eligibility, confirmed eligible, included in the study, completing follow-up, and analysed	NA
	13b	Give reasons for non-participation at each stage	NA
	13c	Consider use of a flow diagram	NA
Descriptive Data	14a	Give characteristics of study participants (eg demographic, clinical, social) and information on exposures and potential confounders	**9**
	14b	Indicate number of participants with missing data for each variable of interest	14
	14c	Cohort study—Summarise follow-up time (eg, average and total amount)	NA
Outcome Data	15*	Cohort study—Report numbers of outcome events or summary measures over time<br>Case-control study—Report numbers in each exposure category, or summary measures of exposure<br>Cross-sectional study—Report numbers of outcome events or summary measures	NA
Main Results	16a	Give unadjusted estimates and, if applicable, confounder-adjusted estimates and their precision (e.g. 95% confidence interval). Make clear which confounders were adjusted for and why they were included	15,16
	16b	Report category boundaries when continuous variables were categorized	NA
	16c	If relevant, consider translating estimates of relative risk into absolute risk for a meaningful time period	NA

SECTION	ITEM NUMBER	CHECKLIST ITEM	REPORTED ON PAGE NUMBER:
	16d	Report results of any adjustments for multiple comparisons	NA
Other Analyses	17a	Report other analyses done—e.g. analyses of subgroups and interactions, and sensitivity analyses	24,25
	17b	If numerous genetic exposures (genetic variants) were examined, summarize results from all analyses undertaken	NA
	17c	If detailed results are available elsewhere, state how they can be accessed	3,4
**DISCUSSION**			
Key Results	18	Summarise key results with reference to study objectives	5
Limitations	19	Discuss limitations of the study, taking into account sources of potential bias or imprecision. Discuss both direction and magnitude of any potential bias	8
Interpretation	20	Give a cautious overall interpretation of results considering objectives, limitations, multiplicity of analyses, results from similar studies, and other relevant evidence	5-8
Generalisability	21	Discuss the generalisability (external validity) of the study results<br>Other information	5-9
**METHODS**			
Study design	4	Present key elements of study design early in the paper	9
Setting	5	Describe the setting, locations, and relevant dates, including periods of recruitment, exposure, follow-up, and data collection	9
Participants	6a	Cohort study—Give the eligibility criteria, and the sources and methods of selection of participants. Describe methods of follow-up<br>Case-control study—Give the eligibility criteria, and the sources and methods of case ascertainment and control selection. Give the rationale for the choice of cases and controls<br>Cross-sectional study—Give the eligibility criteria, and the sources and methods of selection of participants	9
	6b	Cohort study—For matched studies, give matching criteria and number of exposed and unexposed<br>Case-control study—For matched studies, give matching criteria and the number of controls per case<br>Variables	NA

SECTION	ITEM NUMBER	CHECKLIST ITEM	REPORTED ON PAGE NUMBER:
Variables	7	Clearly define all outcomes, exposures, predictors, potential confounders, and effect modifiers. Give diagnostic criteria, if applicable	14-15
Data sources/measurements	8*	For each variable of interest, give sources of data and details of methods of assessment (measurement). Describe comparability of assessment methods if there is more than one group.	9-12
Bias	9	Describe any efforts to address potential sources of bias.	10,14-15
Study size	10	Explain how the study size was arrived at	9
Quantitative variables	11	Explain how quantitative variables were handled in the analyses. If applicable, describe which groupings were chosen and why .	10-13
Statistical methods	12a	Describe all statistical methods, including those used to control for confounding	14-15
	12b	Describe any methods used to examine subgroups and interactions	14-15
	12c	Explain how missing data were addressed	10,14
	12d	Cohort study—If applicable, explain how loss to follow-up was addressed<br>Case-control study—If applicable, explain how matching of cases and controls was addressed<br>Cross-sectional study—If applicable, describe analytical methods taking account of sampling strategy	NA
	12e	Describe any sensitivity analyses	15

*
